# Post COVID – 19 neurological disorders; a single center experience; a case series

**DOI:** 10.1016/j.amsu.2022.103508

**Published:** 2022-03-27

**Authors:** Shwan A. Ahmad, Shvan H. Mohammed, Berwn A. Abdulla, Bestoon Kh Salih, Marwan N. Hassan, Abdulwahid M. Salih, Fahmi H. Kakamad, Hiwa O. Abdullah, Hemn Ali Hassan, Snur Othman, Shadi Hamid Sidiq

**Affiliations:** a Smart Health Tower, Madam Mitterrand Street, Sulaimani, Kurdistan, Iraq; b Kscien Organization, Hamdi Str, Azadi Mall, Sulaimani, Kurdistan, Iraq; c College of Medicine, Univerity of Sulaimani, Madam Mitterrand Street, Sulaimani, Kurdistan, Iraq

**Keywords:** COVID-19, Neurological, SARS-CoV2, Neurology, ARDS

## Abstract

**Introduction:**

Coronavirus disease 2019 (COVID-19) pandemic, is a newly conducted respiratory disease caused by infection with the severe acute respiratory syndrome coronavirus 2 (SARS-CoV2). The current study aims to estimate the neurological diseases which develop after COVID-19 infection.

**Method:**

This is a single center retrospective case series conducted in seven months. the patients were collected in an out-patient clinic. Diagnosis of COVID-19 and the way of diagnosis is confirmed through either polymerase chain reaction (PCR) test for COVID-19 and/or typical findings on chest computed tomography scan (CT scan). Patients developed neurological symptoms after being infected with COVID-19. Symptoms have to be developed within less than 6 months of recovery, or developed during illness and persisted after recovery.

**Result:**

A total number of 59 patients infected with SARS-CoV2 were included. The majority of the patients had mild symptoms 32 (54%), 12 (20%) patients developed severe symptoms. Headache was the most common presenting symptom 27(46%) followed by fatigue in 8 (13.5%). The majority of the patients 55 (91.6%) presented with no focal signs. MRI was done for 27 (46%) patients without abnormal finding in 22 cases. Nearly 22 (37.3%) cases were diagnosed as recurrent episodes of migraine or new onset of migraine. All patients were managed according to the underlying pathology, only (28, 47.5%) patients were known to be completely recovered.

**Conclusion:**

SARS-CoV2 can invade and cause inflammation in the central and peripheral nervous systems. It is responsible for many neurological problems. More studies are necessary to analyze the long term effect of the virus on the nervous system.

## Highlights

• COVID-19 survivors might be at risk of developing neurological disorders.
• The exact prevalence and risk groups are yet to be established.
• This study aims to estimate the post-COVID-19 neurological sequelae.

## 1 Introduction

Coronavirus disease 2019 (COVID-19), is a newly recognized respiratory disease caused by infection with severe acute respiratory syndrome coronavirus 2 (SARS-CoV2) [1]. First, it was discovered in China and in a few weeks became a global pandemic [2]. According to WHO, as of May 10, 2021, it has infected more than 157 million people and caused more than 3.2 million deaths [3]. Angiotensin converting enzyme 2 (ACE2) receptor is the primary target of the virus which is more common in the epithelial cells of the respiratory and gastrointestinal tracts [4]. It is a highly contagious, young aged people may pass the disease asymptomatically, while older and immunocompromised patients are more vulnerable to present with severe symptoms and associated with a higher mortality [5]. The most common and significant manifestation is with respiratory disease, however, neurological features have been reported [6]. The neuroinvasive potential of the virus can be explained by the presence of the receptors in neuron cells [4]. Many reports found approximately one third of infected patients had neurological features such as dizziness and hyposmia [7]. Since the beginning of the pandemic, it has been suggested that survivors might be at an increased risk of developing neurological diseases in the first three months after the infection [8]. One third of COVID-19 patients develop a neurological or psychiatric disorder within six months after infection [9].

As the pandemic persists, a substantial number of individuals infected with the virus may develop short-, medium- and long-term neurological sequelae which have a major impact on everyday activities and quality of life. The current study aims to estimate the neurological diseases that developed after infection with SARS-CoV2.

## 2 Method

### 2.1 Study design

This is a single center retrospective case series conducted in seven months from August 2021 to April 2021. This paper has been written in line with PROCESS 2020 guidelines [10].

Study Registration: The study was registered in Research Registry. The unique identifying number is:: researchregistry7666, and the registration can be found here: Browse the Registry - Research Registry.

### 2.2 Participants

Patients were collected in an out-patient clinic of neurology. Diagnosis of COVID-19 was confirmed through either polymerase chain reaction (PCR) test for COVID-19 and/or typical findings on chest computed tomography scan (CT scan). Accepted ways of diagnosis are: viral PCR, IgG and IgM, and chest CT scan with obvious sign and symptoms of COVID-19.

### 2.3 Inclusion criteria

Patients developed neurological symptoms after being infected with COVID-19. Symptoms had to be developed within less than 6 months of recovery, or developed during illness and persisted after recovery. These symptoms should be clearly related to COVID-19 infection.

### 2.4 Data collection and analysis

The data were received from the center's database. Microsoft excel 2019 was used for collection of the data. Then, the Statistical Package for the Social Sciences (SPSS) Version 25 was used for coding of the data and performing data analysis.

## 3 Results

A total number of 59 patients infected with SARS-CoV2 were included in the study. The age ranged from 16 to 80 years with a mean age of 39.44 years. The majority of cases were female (41, 69.5%). Most of the patients had mild symptoms (32, 54%), severe symptoms were observed in (12, 20%) patients. The median age of severe group was 39.44 years and the mild group was 38.5 years. The duration of COVID-19 varied from 7 to 31 days with a mean of 17.33 days. The duration between negative test for COVID-19 and the onset of symptoms ranged from 3 to 186 days with a mean duration of 73 days. Headache was the most common presenting symptom (27, 46%) followed by fatigue in 8 (13.5%) cases. The majority of the patients (55, 91.6%) had no focal signs. Magnetic resonance imaging (MRI) was done for 27 (46%) patients which was unremarkable in 22 cases. Nearly (22, 37.3%) patients were diagnosed as a new onset of migraine or recurrent episode of migraine, sinusitis in (5, 8.5%), unknown diagnosis in (4, 6.8%), post COVID-19 tension headache in (6, 10%), one patient (1.7%) was diagnosed for each of the following diseases (optic neuritis, Guillain Barre syndrome, vascular dementia, transient ischemic attack, post viral asthenia, mastoiditis, panic attack, vestibular neuritis). All patients were managed according to the underlying pathology, 28 (47.5%) patients were known to be completely improved.

## 4 Discussion

SARS-CoV-2 virus belongs to the family of coronoviridae, which is a large family of the single stranded RNA viruses found in different animal species [11]. Presence of glycoprotein on the envelope gives a crown-like appearance under the electron microscope and facilitates the entry of the virus into the target cells [9,11]. Although the primary route of transmission is by inhalation through respiratory droplets and close contact with infected persons, it is possible to transmit through feco-oral route [12]. The severity of infection with SARS-CoV2 is classified into three categories; patients with mild form experience controllable symptoms and can be treated as outpatients, moderate cases may develop dyspnea and need oxygen therapy at home, and severe cases need hospitalization and intensive care unit (ICU) [13]. Approximately 40 distinct neurological manifestations of COVID-19 have been described in the literature [14].

Patients often suffer from sustained impairment of physical function, cognitive, and psychological impairments over a long period of time after being recovered [15]. In recovered patients, the virus remains for a long period of time in the central nervous system and has the ability to trigger and reactivate neurological complications [14]. The term post-COVID syndrome used for those patients that have a persistent symptom or develop a new condition after being recovered from the disease, the symptoms and conditions are various ranging from minor symptoms such as headache to more critical conditions such as stroke, pulmonary fibrosis, and renal impairment [13]. The syndrome is caused by the residual inflammation, organ damage, nonspecific effects of prolonged ventilation, and social distancing [16]. Among the most common experiencing symptoms after infection are anosmia, ageusia, dysgeusia, headache, musculoskeletal pain and fatigue [15]. As the current study, headache and fatigue are the most common presenting symptoms. According to the previous studies, neurological manifestations in COVID-19 patients are increasing [17].

Although the mechanism of invasion of central nervous system (CNS) by the virus is unknown, SARS-CoV2 may invade CNS by retrograde neuronal transfer through the olfactory bulb [18]. Four pathogenic mechanisms are involved in damaging the CNS such as direct viral encephalitis, systemic inflammation, peripheral organ dysfunction, and change in cerebrovascular system [19]. In some circumstances, the neurological problems may develop from the combination of them [19]. Although no direct link has been established, multiple cases have been reported regarding the association of SARS-CoV2 with chronic CNS disorders such as neurodegenerative disorders, multiple sclerosis, acute flaccid paralysis, Guillain barre syndrome, transverse myelitis and seizure [7,[20], [21], [22], [23]]. The current study reported cases of Guillain barre syndrome, vascular dementia, seizure, and transient ischemic attack. Hyposmia and anosmia are the main neurological abnormalities reported in a three month-follow up [24]. However, new onset of migraine or recurrent episode of migraine was the main neurological problem in this study.

In a multicenter study from Italy, 25 cases of encephalitis associated with SARS-CoV2 were reported with the most common presenting symptoms were delirium, speech disturbance and seizure [25]. Rass et al. reported that a third of patients who admitted to ICU develop encephalitis, with only 3% have persistent features beyond three months [15]. There are evidences showing that the risk of cerebrovascular events is increased post COVID-19, with the increased incidence of ischemic stroke to almost 1 in 10 in encephalopathic patients [8]. The risk of dementia increases by two to three folds following COVID-19 infection [26]. Few studies have been emerged regarding new onset of seizure as a presenting feature in COVID-19 patients [27]. Out of seven cases reported by Anand et al., four cases had a new onset of seizure while the other three cases had a well-controlled epilepsy [27]. Generalized inflammatory polyradiculoneuropathy (Guillain Barre syndrome) preceded by infection with SARS-CoV2 was also reported [25]. The clinical features and the course of the disease were similar to non-COVID-19 patients, the patients received a single course of IVIg and resulted in clinical improvement over 8 weeks [28]. In the present study, one case developed Guillain Barre syndrome and responded well to 5 cycles of plasmapheresis over 4 weeks. Fatigue was reported in approximately 53% of patients with severe COVID-19 after two months from the onset of the symptoms [24]. In the current study, the prevalence decreased to 13%.

Although many studies indicate that SARS-CoV2 can invade CNS and peripheral nervous system (PNS), it is still unclear which patient is at a higher risk for developing neurological complications. Rass et al. reported that severely infected patients have a higher risk of developing persistent neurological disorders 3 months after the infection as compared with the mild and moderate diseases [15]. Nearly 50% of patients who had admitted to ICU developed neurological and psychiatric conditions within six months [9]. However, the relative risk of neurological complications is also increased even in patients with COVID-19 who had only mild symptoms [8]. Compared to flu and other respiratory tract infections, COVID-19 survivors are more vulnerable to develop psychiatric disorders [9]. The current study reported that severe COVID-19 cases are associated with severe neurological impairment such as Guillain Barre syndrome, radiculopathy, migraine and dementia.

There are crucial limitations to this study; the sample size is small, the follow up period is short, and some neurological disorders like cognitive problems are missing.

In conclusion, SARS-CoV2 can invade and cause inflammation in the CNS and PNS through different routes. It remains inside the system and responsible for many short- and long-term neurological problems and may cause irreversible damage to the nervous system. More studies are need to be conducted regarding the long term effect of the virus on the nervous system.

## Provenance and peer review

Not commissioned, externally peer-reviewed.
